# Microscopic Polyangiitis With Pituitary Dysfunction and Spontaneous Renal Aneurysm Rupture

**DOI:** 10.14740/jmc5282

**Published:** 2026-07-28

**Authors:** Shen Ju Liang, Quan You Zheng, Huan Zi Dai

**Affiliations:** a Department of Rheumatology and Immunology, Daping Hospital, Army Medical University (Third Military Medical University), Chongqing 400042, China; b Department of Nephrology and Urology, 958th Hospital, Southwest Hospital, Army Medical University (Third Military Medical University), Chongqing 400020, China; c These authors contributed equally to this work.

**Keywords:** Antineutrophil cytoplasmic antibody, Microscopic polyangiitis, Pituitary dysfunction, Rupture of renal aneurysm, Immunosuppressive therapy

## Abstract

Antineutrophil cytoplasmic autoantibody (ANCA)-associated vasculitis (AAV) is a relatively uncommon autoimmune disease, predominantly causing kidney or lung injury. Pituitary dysfunction is exceedingly rare in microscopic polyangiitis (MPA). Rupture or hemorrhage of renal artery aneurysms is also scarcely reported in AAV, especially in MPA. In this study, we describe a 68-year-old woman who presented with shortness of breath at rest, polydipsia, and polyuria. In addition to an increased p-ANCA titer (1:10) and the presence of anti-myeloperoxidase antibodies, T1-weighted magnetic resonance imaging of the head revealed pituitary injury. A diagnosis of MPA accompanied by pulmonary intestinal disease, central diabetes insipidus, and hypophysitis was confirmed. The patient responded to intravenous methylprednisolone and immunoglobulin. However, 1 week after admission, she complained of a sudden onset of sharp pain in her right waist area, accompanied by weakness, nausea, syncope, and hypotension. The hemoglobin level decreased from 95 to 47 g/L and abdominal computed tomography revealed a large perirenal hematoma surrounding the right kidney. The patient was immediately administered methylprednisolone pulse therapy (500 mg/day for 3 days) and cyclophosphamide (CTX; 0.6 g), followed by fluid resuscitation, blood transfusion, hemostatic therapy, and infusion of fresh frozen plasma. Arterial angiography demonstrated active bleeding of the interlobular artery of the right kidney, and selective arterial embolization was performed. Fortunately, she responded well to glucocorticoid and CTX therapy and did not relapse during the 5-year follow-up. Pituitary involvement accompanied by spontaneous rupture of a renal aneurysm is an extremely rare complication of MPA and has not been reported before.

## Introduction

Antineutrophil cytoplasmic antibody (ANCA)-associated vasculitis (AAV) is a term that describes a group of rare autoimmune diseases characterized by inflammatory cell infiltration and necrotizing damage to small-sized blood vessels [1]. AAV is typically associated with the presence of autoantibodies specific for neutrophil cytoplasmic components (predominantly myeloperoxidase (MPO) and proteinase 3 (PR3)) [2]. As per the definitions established by the International Chapel Hill Consensus Conference in 2012, microscopic polyangiitis (MPA) is mainly related to MPO-ANCA [3].

Central nervous system (CNS) involvement, including the meninges, cerebral vasculature, and pituitary gland, has been reported in 9.8% of patients with MPA [4]. In contrast to several reports of pituitary involvement in granulomatosis with polyangiitis (GPA) [5], only three cases have been reported in MPA [6–8], highlighting the relatively rare occurrence of pituitary dysfunction in the latter condition. Aneurysm formation and rupture of medium- or large-sized vessels are common in polyarteritis nodosa, which is not typically related to ANCA [9]. In AAV, rupture and/or hemorrhage of arterial aneurysms are uncommon events, especially in MPA [10, 11].

Herein, we describe a rare case of MPA accompanied by pituitary dysfunction and spontaneous rupture of a renal aneurysm, which, to the best of our knowledge, has not been reported previously. We also conducted a literature review to better characterize the prevalence, clinical manifestations, pathophysiology, diagnosis, and treatment of MPA with pituitary involvement and renal aneurysms.

## Case Report

### Investigations

In January 2021, a 68-year-old woman gradually developed shortness of breath on exertion, along with cough and expectoration of blood-stained sputum (1–3 mL) 5–10 times daily. The patient complained of polydipsia (consumed over 5 L of water daily) and polyuria, as well as weakness, swelling, and pain in the lower extremities. Oral or vulvar ulcers, rashes, photosensitivity, and hair loss were not present. She received some traditional Chinese medicine but there was no remission. In March 2021, her symptoms worsened, with multiple exudative lesions in both lungs (detected by computed tomography (CT)), and she was administered antibiotics (piperacillin-tazobactam 4.5 g, q8h). Her condition progressively deteriorated, accompanied by glossolalia, shortness of breath at rest, and aggravated pulmonary inflammatory exudation. The patient was admitted to our hospital for further treatment in April 2021. Due to gallstones, she underwent cholecystectomy 10 years ago. She had been diagnosed with hypertension for a decade and irregularly received amlodipine besylate (5 mg daily). She had one daughter, no abortions, and menopause occurred at 47 years of age.

The patient was wheeled into the ward with an acute illness appearance. She was conscious and her vital signs were nearly normal. Apart from slightly moist rales, moderate pitting edema and scattered red petechiae (non-blanching, red, 1–3 mm in diameter) in both lower extremities, other physical examinations yielded insignificant findings.

### Diagnosis

**Figure 1:**
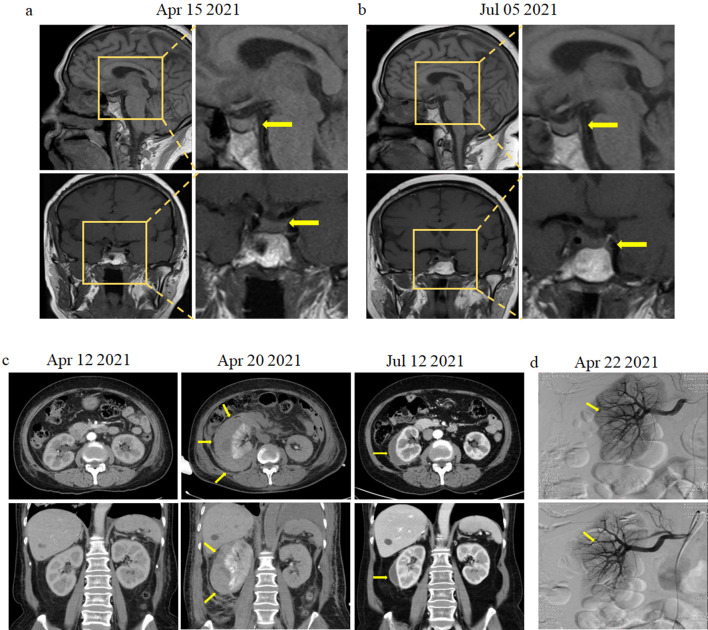
Magnetic resonance imaging (MRI), computed tomography (CT), and arterial angiography images of the patient. (a) Sagittal and coronal pituitary T1-weighted MRI showing local signal enhancement (yellow arrow). (b) Sagittal and coronal pituitary T1-weighted MRI showing normal signal (yellow arrow). (c) Abdominal CT scan showing a large right-sided perinephric and intracapsular hematoma in the right kidney (yellow arrow). (d) Arterial angiography showing active bleeding from the superior segmental and interlobular renal artery of the right kidney. Disseminated arterial microaneurysms are indicated by a yellow arrow (upper panel). Highly selective arterial embolization of the interlobular artery of the right kidney using microcoils (lower panel).

The results of routine laboratory tests revealed a high-level inflammatory response. In addition to the increased p-ANCA titer (1:10) and MPO-ANCA positivity, the hormonal findings showed elevated parathyroid hormone and impaired adrenocorticotropic hormone levels, and a significantly increased aldosterone-to-renin ratio (Supplementary Material 1, jmc.elmerpub.com). Notably, urinalysis indicated low osmolarity and a significant decrease in ion concentration (Supplementary Material 2, jmc.elmerpub.com). Arterial gas analysis suggested hypoxemia and a significantly decreased oxygenation index even with oxygen inhalation (3 L/min). Chest CT scans showed multiple abnormally dense shadows in both lungs, without neoplasm (Supplementary Material 3, jmc.elmerpub.com). Due to the symptoms of polydipsia and polyuria, abnormal adrenocorticotropic hormone level, and low urine osmolarity, she underwent head magnetic resonance imaging (MRI). T1-weighted MRI images (sagittal and coronal) showed morphological changes and local signal enhancement in the sellar region, indicating pituitary injury (Fig. 1a). The result of the water deprivation test was positive, further supporting the diagnosis of central diabetes insipidus (CDI) and hypophysitis. Electronic fiberoptic bronchoscopy detected sticky and foamy secretions with stale hemorrhage in the airway. After suctioning, mucosal congestion was observed in both bronchi at all levels. Lung lavage samples were tested with next-generation sequencing, but no positive results were obtained, which excluded lung infection. The diagnosis of MPA was established based on typical clinical manifestations, positive MPO-ANCA, pulmonary interstitial vasculitis lesions, and pituitary vasculitis injury. The patient fulfilled the 2017 (Draft, score 11: 6 for p-ANCA or MPO-ANCA positivity and 5 for lung fibrosis or interstitial lung disease) and 2022 EULAR/ACR (score 9: 6 for p-ANCA or MPO-ANCA positivity and 3 for lung fibrosis or interstitial lung disease) classification criteria for MPA [12]. Her Birmingham vasculitis activity score (BVAS) was 8 [13].

### Treatment

**Figure 2:**
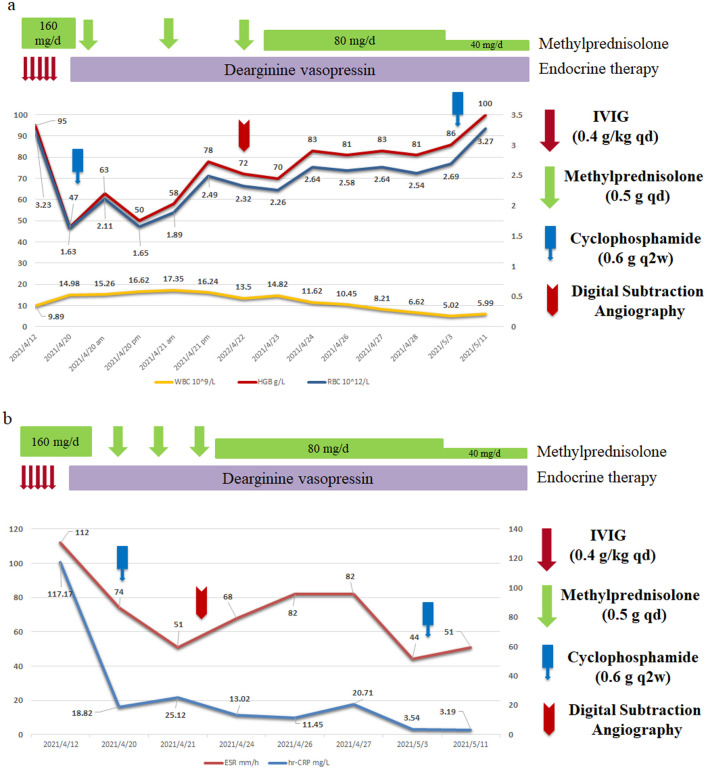
Treatment strategies of this patient. (a) White blood cell (WBC), hemoglobin (HGB), and red blood cell (RBC) response in relation to the serial treatment. (b) Erythrocyte sedimentation rate (ESR) and C-reactive protein (CRP) levels in the serial treatment.

After excluding infection, tuberculosis, and neoplasm, she received intravenous methylprednisolone (160 mg daily), immunoglobulin (0.4 g/kg daily for 5 days), and human albumin (10 g daily), along with multiple supportive therapies, including desmopressin. Owing to the high levels of D-dimer and fibrin, anticoagulant therapy with intradermal injection of low-molecular-weight heparin (0.3 mL, once daily) was administered. Her condition and clinical symptoms gradually improved. One week after admission, the patient complained of a sudden onset of sharp pain in her right waist area, with weakness, nausea, syncope, hypotension, and lethargy. Physical examination revealed anemic appearance and tenderness in the upper right abdomen. The hemoglobin level decreased from 95 to 47 g/L without any obvious blunt trauma. Abdominal CT revealed a large perirenal hematoma surrounding the right kidney (Fig. 1c), indicating renal hemorrhage. We immediately initiated first-line supportive measures including fluid resuscitation, red blood cell transfusion, hemostatic treatment, and fresh frozen plasma infusion. The patient was subsequently administered methylprednisolone pulse therapy (500 mg/day for 3 days) and cyclophosphamide (CTX; 0.6 g), aimed at controlling underlying active MPA vasculitis. Despite receiving a red blood cell suspension (8 units) within 48 h, the hemoglobin level fluctuated between 50 and 78 g/L, and there was no evidence of hemorrhage in other organs, suggesting active bleeding (Fig. 2a). Subsequently, she received arterial angiography. The results of arterial angiography showed a renal artery aneurysm with active bleeding from the superior segmental or interlobular renal artery of the right kidney. Selective arterial embolization of the interlobular artery of the right kidney was then performed using microcoils (Fig. 1d), resulting in stabilization and improvement of hemoglobin levels and clinical symptoms. The patient received intravenous methylprednisolone (80 mg daily) and CTX (0.6 g, every 2 weeks) for active MPA and desmopressin tablets for pituitary dysfunction.

### Follow-up and outcomes

Three weeks after admission, her condition improved, as evidenced by significantly relieved shortness of breath and polydipsia and polyuria. The hemoglobin level improved from 47 to 100 g/L, accompanied by reduced abnormal-density shadows in the lung and ameliorated signs of inflammation (C-reactive protein (CRP) and erythrocyte sedimentation rate (ESR)) (Fig. 2b). The patient was discharged after the third CTX infusion and was treated with prednisolone (50 mg, once daily). During follow-up, multiple chest CT examinations revealed that the pulmonary lesions were gradually absorbed (Supplementary Material 3, jmc.elmerpub.com, and the signal of the pituitary gland (on MRI) had completely recovered (Fig. 1b). Considering the normalized urine osmotic pressure, urine ion levels, and decreased urine volume, desmopressin was gradually discontinued (Supplementary Material 2, jmc.elmerpub.com). Following 12 cycles of CTX (7.0 g in total), MPO-ANCA became negative, and immunotherapy was continued with methotrexate (12.5–15 mg/week). Prednisone was used to maintain remission, and it was gradually tapered from 40 to 5 mg per day. The patient’s BVAS score decreased from 8 to 0, and she has been stable without any specific symptoms during the 5-year follow-up.

### Review of the literature

**Figure 3:**
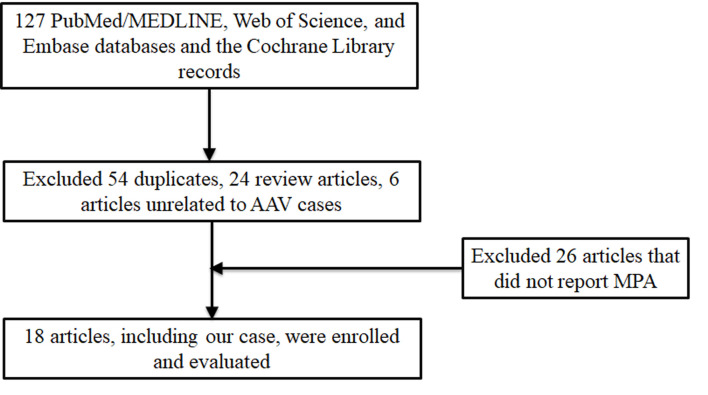
Flow diagram of records in literature review process.

We conducted a systematic literature review by searching the PubMed/MEDLINE, Web of Science, and Embase databases and the Cochrane Library using the following Medical Subject Headings (MeSH) terms: “ANCA-associated vasculitis” or “vasculitis” or “microscopic polyangiitis” AND “hypophysitis” or “pituitary dysfunction or disorders” or “diabetes insipidus” AND “renal rupture” or “kidney/renal aneurysms” or “aneurysms rupture,” including their abbreviations. There were no limitations on publication dates, and only articles available in English were included. We found 127 articles and excluded 54 duplicates, 24 review articles, six articles unrelated to AAV cases, and 26 articles that did not report MPA. Finally, 18 articles, including our case, were included and evaluated (Fig. 3). Of these, three cases of MPA with pituitary dysfunction have been reported (Table 1), in contrast to dozens of relevant GPA cases [6–8]. In addition, 14 cases of MPA with renal aneurysm rupture have been described (Table 2) [10, 11, 14–24].

**Table 1:** Reported Cases of Pituitary Dysfunction in Patients With MPA

Items	Li/2020 [6]	Funauchi/2002 [7]	Mercedes/2025 [8]	This case
Sex/age	M/61	F/62	F/41	F/59
Auto-antibody	MPO-ANCA	MPO-ANCA	MPO-ANCA	MPO-ANCA
Onset of pituitary injury	Onset of MPA	2 months before MPA diagnose	2 weeks before MPA diagnose	Onset of MPA
Symptoms of Pituitary injury	Polydipsia, polyuria, and erectile dysfunction	Polydipsia, polyuria	Persisted headache, sinus/facial discomfort	Polydipsia, polyuria
Imaging findings	MRI (negative), PET-CT: increased linear radioactivity uptake in pituitary fossa	MRI: swelling of the pituitary gland and loss of the high intensity signal of posterior lobe	MRI: enlarged heterogeneous pituitary gland with infundibular thickening	MRI: enlargement of pituitary gland and local signal enhancement
Treatment	PD-GC and HD-GC + CTX	Prednisolone for 4 weeks	GC and rituximab	PD-GC and HD-GC+CTX
Hormone replacement	Desmopressin	Desmopressin	None	Desmopressin
Outcomes	Partial remission	Remission	Remission	Remission

ANCA: antineutrophil cytoplasmic antibody; CTX: cyclophosphamide; F: female; HD-GC: high dose glucocorticoid; M: male; MPA: microscopic polyangiitis; MPO: myeloperoxidase; MRI: magnetic resonance imaging; PD-GC: pulse dose glucocorticoid; PET-CT: positron emission tomography-computed tomography.

**Table 2:** Reported Cases of Renal Aneurysm in Patients With MPA

Case/year	Sex/age	Auto-antibody	Presentation	Imaging examinations	Involved artery	Perirenal hematomas	Treatment	Outcomes
Inatsu/2002 [14]	M/78	MPO-ANCA	Slight fever and weight loss, RPGN	Arterial angiography	Bilateral renal artery	No	Steroids	Recovered, HD
Tamei/2008 [11]	M/55	MPO-ANCA	Sharp pain on the left side, shock	Abdominal CT and arterial angiography	Left renal, hepatic artery	Yes, left	Blood transfusion, steroids	Recovered, HD
Dhaun/2013 [15]	M/56	MPO-ANCA	Bilateral loin pain	CT and CTA	Bilateral renal, hepatic, mesenteric artery	Yes, bilateral	Steroids + CTX, PE	Recovered
Jira/2013 [16]	M/56	MPO-ANCA	Severe right iliac fossa pain hemodynamic shock	Renal arteriography	Bilateral renal artery	Yes, right	IV MP + CYC, oral steroids	Recovered
Nakashima/2015 [24]	F/77	MPO-ANCA	RPGN, both flank pain	Abdominal CT and ultrasonography	Bilateral renal artery	Yes, bilateral	Oral steroids	Recovered, HD
Ishiwatari/2018 [10]	F/82	MPO-ANCA	RPGN, decreased urinary volume, decreased hemoglobin	Abdominal CT	Right renal artery	Yes, right	Coil embolization, IV MP, oral steroids mizoribine	Recovered, HD
Yu/2018 [17]	M/61	MPO-ANCA	Left lumbago, decreased hemoglobin	Contrast enhanced CT	Bilateral renal, hepatic, and mesenteric artery	Yes, left	Nephrectomy in emergency	NA
Zhang/2019 [18]	F/57	MPO-ANCA	Severe lumbago, acute renal failure, and hemorrhagic shock	Abdominal CT	Bilateral renal artery	Yes, bilateral	IV MP, oral steroids	Recovered
Andrew/2020 [19]	F/50	MPO-ANCA	Abdominal pain, decreased hemoglobin	Abdominal CT, contrast enhanced CT	Bilateral renal, hepatic artery	Yes, bilateral	Percutaneous drainage of hematoma, IV MP and CYC	NA
Nunes/2022 [20]	M/52	Negative (kidney biopsy)	Severe abdominal pain and hemorrhagic shock	CTA	Left renal artery	Yes, left	Coil embolization, IV MP + CYC, oral steroids	Recovered
Zhao/2023 [21]	F/56	MPO-ANCA	Back pain	Abdominal CT	Right renal artery	Yes, right	Oral steroids + CTX, PE	Recovered
Tang/2024 [22]	M/61	MPO-ANCA	Low back pain, decreased hemoglobin	Renal ultrasonography, arteriography	Bilateral renal artery	Yes, bilateral	IV MP + CYC, PE and coil embolization	Recovered, HD
Jana/2025 [23]	F/45	MPO-ANCA	Severe abdominal pain, drop in hemoglobin	Abdominal CT, MRI, and CTA	Left renal artery	Yes, left	IV MP + CYC, oral steroids	Recovered
This case	F/59	MPO-ANCA	Sharp pain in her right waist area, hypotension, decreased hemoglobin	Abdominal CT, contrast enhanced CT, arteriography	Right renal artery	Yes, right	Coil embolization, IV MP + CYC, oral steroids	Recovered

ANCA: antineutrophil cytoplasmic antibody; CT: computed tomography; CTA: computed tomography angiography; CTX: cyclophosphamide; F: female; HD: hemodialysis; IV MP: intravenous methylprednisolone; M: male; MPO: myeloperoxidase; NA: not available; PE: plasm exchange.

## Discussion

MPA, a devastating and potentially lethal form of autoimmune disease, primarily manifests as necrotizing glomerulonephritis and pulmonary capillaritis [1, 2]. It frequently occurs at the age of 60–65 years, with a slight male prevalence [2]. The CNS is affected in less than 15% of patients with AAV and in fewer with MPA (10%) [25]. Pituitary involvement is a relatively rare clinical manifestation in AAV and extremely rare in MPA, with only three cases reported [6–8]. It was shown that the clinical symptoms of pituitary injury could occur before and simultaneously with MPA (Table 1), which is consistent with the notion that pituitary dysfunction can occur at any time in AAV [5, 6]. CDI and hypogonadotropic hypogonadism tend to be prominent manifestations of pituitary dysfunction in AAV, followed by hypothyroidism and hypogonadism [26]. Liu et al [25] reported a frequency of CDI up to 85.7% in GPA with pituitary disorder, and initial symptoms in more than 35% of patients included polyuria, polydipsia, decreased libido, and other constitutional symptoms, such as headache, weakness, lethargy, appetite loss, and vomiting. Consistent with this, we also found that polydipsia and polyuria were the major initial symptoms of pituitary involvement in patients with MPA (Table 1). Visual deficits can occur if the optic chiasm is compressed by pituitary enlargement [27]. Moreover, hyperprolactinemia can occur when the pituitary stalk is impaired by inflammatory vasculitis, manifesting as galactorrhea [28]. Accordingly, early awareness of AAV as one of the underlying causes of pituitary dysfunction is crucial for physicians, especially neurologists.

The underlying pathophysiological mechanism of pituitary dysfunction in AAV remains elusive [6].

Pathogenic ANCAs targeting PR3 and MPO in innate immune cells, especially neutrophils, are induced by multiple genetic, environmental, and immunological factors [1]. ANCAs are the major contributors to the initiation and progression of AAV [2]. Following interaction with their specific antisense peptides, PR3 or MPO triggers an uncontrolled immune self-amplification network, leading to the two major pathological processes in AAV, vasculitis and granulomatosis [2, 29]. Neutrophils, activated by ANCAs and enhanced by components of the alternative complement, are pivotal in the pathology of vasculitis. Upon activation, neutrophils can transmigrate across the vessel wall and promote degranulation, oxidant respiratory burst, apoptosis, and necrosis, triggering disruption of the endothelium, a coagulation cascade, and fibrinoid necrosis of small- or medium-sized vessels [1]. This process is further enhanced by the alternative complement pathway, with the C3-C5 axis playing a critical role [30]. As to pituitary disorders in AAV, the following pathologic processes should be taken into consideration: (1) systemic vasculitis caused by inflammation, obstruction, or increased permeability of the affected vessels; (2) infiltration or compression by granulomatosis from adjacent structures; or (3) *de novo* granulomatous formation in the pituitary system [5, 6]. In MPA, systemic vasculitis may account for the major pathological mechanism owing to the absence of granulomatous inflammation, which requires further investigation.

Although testing for ANCA, a valuable biomarker for diagnosing ANCA-related vasculitis, has 98.5% specificity and 96% sensitivity for AAV [31], negative serological results should not exclude an AAV diagnosis, as 10% of patients with AAV lack ANCAs [32]. In particular, ANCA-negative results can be produced in AAV without kidney or lung lesions [33]. Visual assessment may be a meaningful initial investigation, considering that nearly 40% of patients with AAV experience visual deficits [26]. Radiological examinations, especially MRI, are essential for confirming pituitary gland involvement, and MRI abnormalities are found in 94.1% of patients [34], with frequent findings of an enlarged pituitary gland and sellar mass (in 77.9%), absence of posterior hypersignal on T1-weighted images (in 42.6%), and thickening or abnormal enhancement at the pituitary stalk (in 17.6%) [26]. Negative MRI results should not exclude pituitary involvement. Li et al [6 ] reported that MPA with pituitary dysfunction showed increased linear radioactivity uptake on positron emission tomography (PET)/CT compared with negative MRI findings, indicating the potential application of PET/CT in the diagnosis of pituitary dysfunction [35]. Pituitary biopsy is the gold standard for confirming the diagnosis, despite the potential risk of surgery [36]. Owing to multiple organ involvement in AAV, tissue sampling can be performed at safer sites, but a pituitary biopsy might be required under the following conditions: (1) exclusion of a differential diagnosis of pituitary lesions; (2) challenging diagnosis of pituitary dysfunction; and (3) progression during regular immunosuppressive treatment and in cases of relapse [27]. In summary, AAV should be considered in the differential diagnosis of unexplained pituitary dysfunction accompanied by existing systemic manifestations. Specifically, ANCA testing, head MRI, PET/CT analysis, and pituitary biopsy are extremely meaningful approaches for confirming the diagnosis.

Given its extreme rarity, with three cases reported to date, treatment strategies for pituitary dysfunction in patients with MPA are mostly based on those for GPA-related pituitary dysfunction [25]. In addition to hormonal replacement, treatment of ANCA-associated pituitary dysfunction often involves a combination of long-term glucocorticoid and immunosuppressants such as CTX, methotrexate, azathioprine, and rituximab (RTX) [1 ]. High-dose glucocorticoid combined with CTX is most commonly used, reducing 1-year mortality in GPA from 80% to 10–20% [27]. RTX, a specific B lymphocyte-depleting anti-CD20 monoclonal antibody, indirectly reduces circulating ANCA by depleting B-cell progenitors of ANCA-secreting plasma cells, rather than directly eliminating ANCA itself. A landmark clinical trial confirmed that its therapeutic effect is equivalent to standard induction therapy (glucocorticoid and CTX), with comparable remission rates at 6 months [37]. Recently, RTX was recommended as a first-line therapy for severe AAV, although specific clinical trials on rare manifestations such as pituitary dysfunction are lacking [38]. The C5a-receptor antagonist avacopan is a novel glucocorticoid-sparing regimen for AAV management [39], and, when combined with RTX or CTX, showed similar efficacy to glucocorticoid treatment in an essential randomized controlled trial [40]. Consistent with this, all patients received a combination of long-term glucocorticoid and immunosuppressants for the treatment of MPA-associated pituitary dysfunction, including one treated with RTX (Table 1), suggesting classic treatment protocols are also suitable for pituitary disorders in MPA [8 ].

Renal artery aneurysm formation and rupture are rare in MPA [41]. To the best of our knowledge, 14 cases with detailed clinical information, including this case, have been reported (Table 2). The median age was 60 (45–82) years, and half of these patients were male. The main symptoms or signs of aneurysms rupture were acute abdominal pain and hemorrhagic shock. Imaging examinations for renal aneurysm mainly involved abdominal CT (50%), contrast-enhanced CT (16%), CT angiography (16%), and catheter angiography (27%), suggesting CT is a powerful diagnostic examination for rupture of renal aneurysms in MPA. Almost all patients were positive for MPO-ANCA, except one, who was diagnosed by biopsy, supporting the idea that negative serological results should not exclude an MPA diagnosis. Four cases (22%) also had hepatic or mesenteric artery involvement. Moreover, eight cases (44%) had bilateral renal artery aneurysms, and five of them (63%) suffered from bilateral perirenal hematomas, indicating multiple aneurysm formation and rupture in MPA.

Aneurysms of medium-sized arteries are more commonly encountered in polyarteritis nodosa, while their rare occurrence in smaller arteries precludes any definitive conclusions [10, 41]. Renal aneurysms in MPA are characterized by the formation of microaneurysms that mostly affect interlobular arteries and are sausage-shaped [42]. The pathogenesis of microaneurysms remains unclear, with some hypotheses partly accounting for their formation and rupture. Firstly, activated neutrophils transmigrate across the vessel wall and promote oxidative respiratory burst, degranulation, apoptosis, and necrosis, causing fibrinoid necrosis of small- to medium-sized arterial walls. This process may disrupt the internal and/or external elastic lamina, ultimately contributing to the initiation and progression of aneurysmal dilation [41]. Additionally, ANCA-induced vasculitis can weaken the vessel wall and promote aneurysmal development, as observed in large-vessel vasculitis [10]. Thirdly, arterial narrowing and anticoagulant therapy for thrombosis, malignancy, and atherosclerosis cannot be completely ruled out [10, 11]. Lastly, classic immunosuppressive strategies always lead to adventitial thinning [43]. Five cases (28%) were diagnosed with MPA and renal aneurysms simultaneously. The remaining cases, who received immunosuppressive or anticoagulant therapies, were diagnosed with renal aneurysm within 30 days. These findings suggest that immunosuppressive and anticoagulant therapies might have partly accounted for the spontaneous rupture of the renal aneurysm, and patients with MPA undergoing such therapies should pay more attention to renal aneurysm rupture. Research on the underlying pathophysiological mechanisms of renal aneurysm formation in AAV is expected to inform targeted therapy strategies.

Immunosuppression may be considered if vasculitis is determined to be the main cause [1 ]. Routine immunosuppressive strategies, including long-term glucocorticoid and immunosuppressants, may be indicated when AAV is activated. All patients responded well to the combined therapies, including five (28%) undergoing maintenance hemodialysis. Moreover, treatment of ruptured renal artery aneurysms in patients with AAV includes supportive therapies, especially fluid resuscitation and blood transfusion [10]. When active bleeding is suspected, selective arterial angiography and coil embolization should be performed in a timely manner. Four patients underwent selective arterial angiography and coil embolization to control persistent bleeding of the renal aneurysm. Additionally, one patient underwent emergency nephrectomy [17], and another underwent percutaneous drainage of the hematoma. In emergency situations, surgical intervention is an important measure for treating renal aneurysm rupture, especially in patients with uncontrolled bleeding [17, 19]. Fortunately, our patient responded well and rapidly achieved AAV remission after three cycles of glucocorticoid pulse therapy (0.5 g per day) and several rounds of CTX infusion. Maintenance therapies included methotrexate (12.5–15 mg/week) and tapered prednisone (tapered from 50 mg to 5 mg per day), and there was no relapse during the following 5 years.

### Learning points

Pituitary dysfunction can occur at any time during the complex course of MPA and may even present as a primary manifestation. CDI and hypogonadotropic hypogonadism are common endocrine features in AAV with pituitary involvement, and AAV should be considered in the differential diagnosis.

MPA with rupture of a renal aneurysm and active bleeding is a rare but urgent clinical condition with high mortality. Highly selective renal artery embolization is the preferred therapeutic option for such patients.

Systemic immunosuppressive therapies should be started at an early stage to induce MPA remission.

## Supplementary Material

Suppl 1 Laboratory examinations of the patient when administration.

Suppl 2 Urinalysis of the patient before and after immunosuppressive therapies.

Suppl 3 Images of chest computerized tomography scan during treatment. During follow-up, multiple chest CT examinations revealed that the pulmonary lesions were gradually absorbed.

## Data Availability

The data supporting the findings of this study are available from the corresponding author upon reasonable request.
