# Supplementary material for: Microscopic Polyangiitis With Pituitary Dysfunction and Spontaneous Renal Aneurysm Rupture
Source: J Med Cases. 2026 Jul 28;17(9):439–48. doi: 10.14740/jmc5282 (PMC13442982; doi:10.14740/jmc5282)
Supplement: Supplementary file 1 [file jmc-17-09-439-s001.docx]

Suppl 1. Laboratory examinations of the patient when administration.

| Value | Normal range | Results | Value | Normal range | Results |
| --- | --- | --- | --- | --- | --- |
| WBC | 3.8-9.5 (×10^12/L) | 9.89 | IL-6 | 0-7 (pg/ml) | 254.5 |
| RBC | 3.8-5.1 (×10^12/L) | 1.9 | PCT | 0-0.25 (ng/L) | 0.95 |
| HB | 115-150 (g/L) | 95 | Serum K^+^ | 3.5-5.5 (mmol/L) | 3.62 |
| PLT | 94-268 (×10^9/L) | 463 | Serum Ca^2+^ | 2.11-2.52(mmol/L) | 2.03 |
| Neutrophil (%) | 40-75 | 84 | Vitamin D | ＞ 30.00 (ng/ml) | 17.63 |
| Lymphocyte (%) | 20-50 | 9.8 | Anti-ANA | Negative | Negative |
| TBiL | 1.7-23.0（μmol/L） | 25.3 | Anti-MPO | Negative | +++ |
| DBiL | 0.00-6.8（μmol/L） | 14.3 | Anti-pANCA | Negative | 1:10 |
| D-dimer | 0-232 (μg/L) | 10151.2 | Anti-CCP | Negative | Negative |
| Fibrin | 2-4 (g/L) | 7.46 | RF | Negative | Negative |
| AST | 13.0-35.0 (U/L) | 225.8 | ARR | < 3.7 （ng/dl/μlU/mL） | 16.72 |
| ALT | 7.0-40.0 (U/L) | 191.7 | PTH | 12-28（pg/mL） | 164.97 |
| ALB | 40.0-55.0 (g/L) | 26.5 | ACTH | 4.7-48.8（pg/mL） | 4.31 |
| Urea nitrogen | 2.6-7.5 (mmol/L) | 9.58 | CORT | 185-624 (nmol/L) | 219.6 |
| Crea | 41.0-97.0 (μmol/L) | 84.5 | FT3 | 3.09-7.42 (pmol/L) | 3.56 |
| Cys-C | 0-1.26 (mg/L) | 2.16 | HSV IgM |  | + |
| eGFR | >90 ml/min/1.73m^2^ | 76.13 | T-SPOT |  | Negative |
| Complement C3 | 0.79-1.52 (g/L) | 1.29 | Hepatitis virus |  | Negative |
| Complement C4 | 0.16-0.38 (g/L) | 0.24 | EB virus |  | Negative |
| hr-CRP | 0-8 (mg/L) | 117.17 | TP |  | Negative |
| ESR | 0.0-20 (mm/h) | 112 | HIV |  | Negative |
