# Supplementary material for: Microscopic Polyangiitis With Pituitary Dysfunction and Spontaneous Renal Aneurysm Rupture
Source: J Med Cases. 2026 Jul 28;17(9):439–48. doi: 10.14740/jmc5282 (PMC13442982; doi:10.14740/jmc5282)
Supplement: Supplementary file 2 [file jmc-17-09-439-s002.docx]

Suppl 2. Urinalysis of the patient before and after immunosuppressive therapies.

| Value | Apr 16 2021 | Jun 15 2021 | Normal range |
| --- | --- | --- | --- |
| Osmotic pressure | 360 **↓** | 600 | 600-1000 (mOsm/L) |
| Specific gravity | 1.008 | 1.010 | 1.005-1.030 |
| U-Uric | 1.13 **↓** | 2.58 | 2.4-6.0 (mmol/L) |
| U-K^+^ | 19.62 **↓** | 30.19 | 25-100 (mmol/L) |
| U-Na^+^ | 18.6 **↓** | 143 | 130-260 (mmol/L) |
| U-Cl^-^ | 19.4 **↓** | 139.9 | 170-250 (mmol/L) |
| U-Ca^2+^ | 0.09 **↓** | 2.71 | 2.5-7.5 (mmol/L) |
| U-P | 8.99 | 9.02 | 23-48 (mmol/L) |
| U-Mg^2+^ | 0.21 **↓** | 2.86 | 2.0-8.0 (mmol/L) |
| Protein | Negative |  | Negative |
| RBC | Negative |  | Negative |
| Bilirubin | Negative |  | Negative |
| Urine culture | Negative |  | Negative |
