# Supplementary material for: Microscopic Polyangiitis With Pituitary Dysfunction and Spontaneous Renal Aneurysm Rupture
Source: J Med Cases. 2026 Jul 28;17(9):439–48. doi: 10.14740/jmc5282 (PMC13442982; doi:10.14740/jmc5282)
Supplement: Supplementary file 3 [file jmc-17-09-439-s003.docx]

Suppl 3. Images of chest computerized tomography scan during treatment. During follow-up, multiple chest CT examinations revealed that the pulmonary lesions were gradually absorbed.
